# Anæsthetics for Children

**Published:** 1878-12

**Authors:** 


### EDITORIAL, ETC.

*Anæsthetics for Children.*—Probably no more distressing sight is often presented to the dentist than that of a nervous, timid, crying child, suffering with a raging tooth-ache; often with but little sympathy from those who accompany it, and frightened at the idea—a dread which seems instinctive—of an operation. With nerves wrought up to their highest tension, and brain all excited by pain and loss of sleep, the little sufferer is indeed a pitiable object. No persuasions avail to overcome the sense of blind fear, (for this terror is not unreasonable but unreasoning) and the whole spectacle is one of worry, distress and delay. The idea of force is so repugnant to the finer sensibilities as not to be thought of; and dentist and parent are apt to lose temper. He whose heart does not sympathize with the child should not have the care of children, and yet the offending member must be removed.

We have found for years past in chloroform an agent exceedingly useful in such cases, and they are nearly the only cases in which it is used by us. The susceptibility of young children to this anæsthetic, its innocuous effects, the small quantity necessary, and their prompt recovery, point it out as too valuable to be dispensed with. Struggles and screaming only facilitate its administration, as the deep inspirations incident to crying enable the drug to reach the deepest parts of the lungs; and it is surprising how small a quantity inhaled brings on unconsciousness. "Babies take chloroform as they do milk," says an authority, and it would seem that this agent, over which there is so much dispute as to the safety of, in adult use, is harmless with children. If we can save them a pang, it is our duty to do so, and that chloroform is not only a help in this respect but an advantage to the dentist in such cases, avoiding struggling and surgical accidents, we are quite sure. H.

